# Ossifying odontogenic fibroma: A rare case report

**DOI:** 10.4317/jced.51440

**Published:** 2014-12-01

**Authors:** Márcio-Bruno-Figueiredo Amaral, Giovanna-Ribeiro Souto, Martinho-Campolina-Rebello Horta, Ricardo-Santiago Gomez, Ricardo-Alves Mesquita

**Affiliations:** 1 Department of Oral Surgery and Pathology. School of Dentistry. Universidade Federal de Minas Gerais. Belo Horizonte - MG, Brazil; 2 Department of Dentistry, Pontifícia Universidade Católica de Minas Gerais. Belo Horizonte - MG, Brazil

## Abstract

Odontogenic fibroma (OF) is a rare benign odontogenic neoplasm that is most commonly found in the mandibular/premolar region of female patients in the second to fourth decades of life. Well-defined radiolucent lesions that may induce root resorption are normally observed. Rare variants of OF have been described in the prior literature, including references to: 1) giant cell lesions, 2) amyloid-like protein deposition, and 3) ossifying variants. Immunohistochemistry can contribute to understanding the biological behavior and the pathogenesis of OF. Therefore, this case report aimed to describe a new case of ossifying OF and discuss the histopathology and immunohistochemical features.

** Key words:**Odontogenic fibroma, jaw tumors, ossifying variant.

## Introduction

Odontogenic fibroma (OF) is a rare benign odontogenic neoplasm. Clinically, OF is most common in the mandibular/premolar region of female patients in the second to fourth decades of life and is frequently found as radiolucent lesions that may induce root resorption (1).

Histopathologically, OF is characterized by variable amounts of inactive-looking odontogenic epithelium embedded in a mature ﬁbrous connective tissue stroma (2). Although odontogenic epithelial rests does not define the diagnosis, these structures must be observed to reach a diagnosis of OF (3).

The cytokeratin (CK) expression may well be a useful tool to conﬁrm the diagnosis of OF in epithelium-poor cases and to reveal the main types expressed in the OF when compared to other odontogenic tumors (3). Rare variants of OF have been described in prior literature including references to: 1) with giant cell lesions, 2) amyloid-like protein deposition, and 3) ossifying variant (4).

Therefore the present study is aimed to describe a rare case of ossifying odontogenic fibroma and discuss the histopathology and immunohistochemical features of this rare variant of OF.

## Case Report

**Figure 1:**
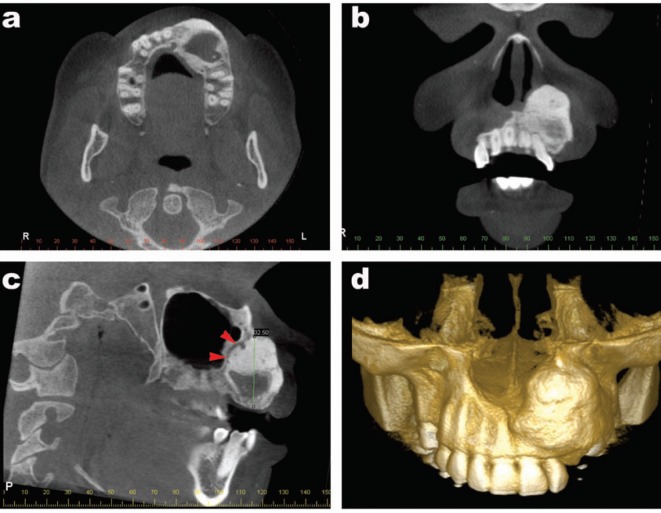
Cone beam computed tomography (CBCT) features of ossifying variant of odontogenic fibroma. a) Axial plane showing a mixed hyperdense-hypodense image with buccal expansion in the left maxilla. b) Coronal plane showing root resorption of teeth 21 and 22. c) In the parasagital plane, a mixed image measuring 32.5 mm associated with a hypodense line (red arrows red) delimiting the lesion of the health bone. d) 3D-CBCT showing buccal expansion of the lesion associated with invasion of the nasal cavity.

**Figure 2:**
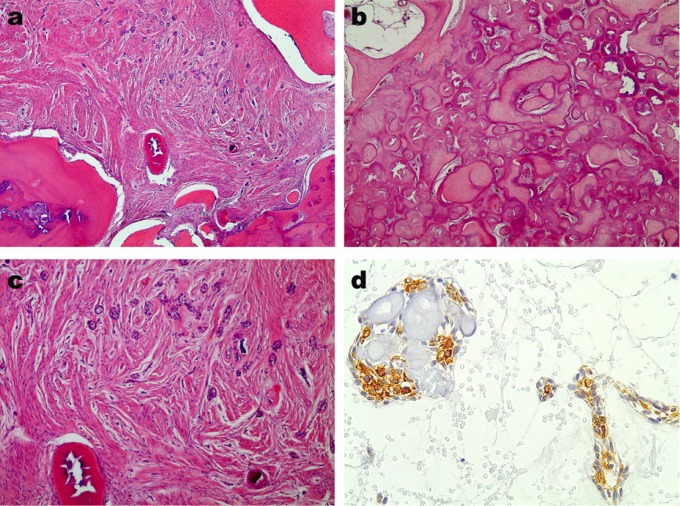
Histopathological and immunohistochemical features of ossifying variant of odontogenic fibroma. a) odontogenic fibroma demonstrates cellular fibroblastic connective tissue with numerous islands and cords of odontogenic epithelium. Reactive bone formation involves the lesion (haematoxylin and eosin (HE staining, 40x original magnification). b) Abundant calcified structures and presence of surrounding conjunctive tissue. c) Islands and cords of odontogenic epithelium, fibroblastic connective tissue and bone formation (HE, 100x original magnification). d) Immunohistochemical staining positive for CK14 in islands and cords of odontogenic epithelium (streptavidin-biotin, 400x original magnification).

A 43-year-old woman with noncontributory medical, social, and cultural records was referred to the Oral and Maxillofacial Surgery Service, Department of Dentistry, Pontifícia Universidade Católica de Minas Gerais in Belo Horizonte, Brazil, to evaluate a nodule in her mouth. The patient reported that the lesion had been presenting a slow progressive growth for 3 years. Through clinical examination, a swelling of the left paranasal area with asymmetry could be observed extraorally. Intraorally, a swelling of the left maxilla associated with teeth 21, 22, and 25 could be identified. Teeth 23 and 24 were not presented. The lesion was painless, uneventful, well-defined, firm consistency, covered by normal color and texture of mucosa, and measured 32 x 30 mm. The cone-beam computed tomography (CBCT) of the maxilla showed a well-defined mixed hyperdense/hypodense lesion (Fig. 1a,c). Expansion of the buccal cortical bone (Fig. 1a,d), as well as root resorption on teeth 21 and 22 (Fig. 1b), could be observed. Additionally, expansion of the lateral wall, associated with invasion of the nasal cavity, could be identified by CBCT imagery (Fig. 1a,c). Ossifying fibroma, calcifying epithelial odontogenic tumor, calcifying cystic odontogenic tumor, complex odontoma, dentinogenic ghost cell tumor, and adenomatoid odontogenic tumor were proposed as differential clinical-radiographic diagnoses. An incisional biopsy was performed and a suggestive histopathological diagnosis of the ossifying fibroma was proposed. Enucleation of the lesion was performed by intraoral approach. An important surgical finding was the easy detachment of the lesion from the healthy bone. Microscopical analysis of surgical piece demonstrated a lesion with fibroosseous features, comprised of composed by mature collagenous fibers and spindle-shaped fibroblasts, with numerous islands and cords of odontogenic epithelium (Fig. 2a,c). This component was intimately admixed with calcified tissue characterized by trabeculae of bone and round shaped structures (Fig. 2b). Immunohistochemical study was performed by employing anti-CK antibodies. Positivity to CK 14 and 19, as well as negativity to CK 7, 8, and 18, could be observed (Fig. 2d). The diagnosis of ossifying OF was therefore established. The postoperative period proved to be uneventful. The patient is currently undergoing routine follow-up with no signs of recurrence after 12 months.

## Discussion

OF is a very rare benign odontogenic tumor. According to the prior literature, OF represents 0 to 5.5% of all odontogenic tumors (3). Although OF has been identified in a wide range of ages, it is most frequently diagnosed in patients between the second to fourth decades of life (5). There is a female predilection for female patients with a female/male ratio of 2.8/1 (2). These data are in accordance with the current case report, which occurred in a 43 year-old female patient. of 43 years of age.

Topographically, OF more commonly affects the premolar region of the mandible and the anterior region of the maxilla (5,6). Clinically, OF is characterized by an asymptomatic slow growth, which results in cortical expansion (7). In the present case, the lesion was found in the anterior region of the maxilla with asymptomatic growth and cortical expansion, as reported in prior literature.

Considering only cases of ossifying OF in prior literature, three cases were more frequently reported in female patients in the fourth decade of life (4,8).

Conventional radiographs, fan-beam computed tomography or CBCT of OF predominantly shows unilocular or multilocular radiolucent images with well-defined margins (9,10). Ikeshima and Utsumomiya (2005) (11) attempted to show specific characteristics of OF for differential diagnoses, such as well-defined limits, root resorption, and calcification and demonstrated varying characteristics for points such as shape, inner lesion, boundary, bone expansion, and root resorption.

Specific radiographic features for OF have yet to be clarified; however, severe effects on the surrounding bone trabeculae are often seen, even if the borders are well-defined in radiographic images. Diagnosis of OF should be complicated by several reasons, such as the diversity of radiographic and histopathological characteristics (5). Moreover, the occurrence of calcified material can produce a mixed radiolucent/radiopaque image (2). Therefore, differential diagnosis of ossifying odontogenic fibroma should be made considering benign tumor lesions with these specific image findings such as ossifying fibroma, calcifying epithelial odontogenic tumor, calcifying cystic odontogenic tumor, complex odontoma, dentinogenic ghost cell tumor, and adenomatoid odontogenic tumor. Ossifying fibroma should be considered as the main differential diagnosis due to similar fibroosseous mixed radiographic findings found in some cases of OF that could induce a misdiagnosis. Furthermore, large lesions can induce root resorption in the involved teeth and cortical expansion (12). In the current case report, a well-defined exuberant hyperdense image could be observed around the hypodense area. In addition, a hypodense line could be observed around the lesion which may well explain the easy detachment of the lesion from the healthy bone. As reported in prior literature, root resorption and bone expansion were also present.

Histologically, OF is defined as an odontogenic tumor consisting of a moderately cellular fibrogenic proliferation with mono-morphic fibroblastic cells and a thin mature collagen admixed with epithelial cords and islands of odontogenic origin (2). According to findings from Mosqueda-Taylor *et al.* (3), the immunohistochemical analysis of the epithelial component of the reported OF demonstrates positivity for CK14 and CK19, and negativity for CK7, CK8, and CK18. Studies in human enamel organ, as well as in the remnants of the dental lamina, showed that these epithelial cells are positive for CKs 7, 13, 14, and 19, and proved negative for CKs 8, 10, 16, 17, and 18 (13). The remnants of the dental lamina are specifically labeled for CK 7, while the lack of CK7 in some calcifying epithelial odontogenic tumors appears to be related to tumoral indifferentiation (14). Differences in concerning specific state of differentiation were observed only for CKs 14 and 19 in the inner enamel epithelium. An intense immunostaining for CK 14 was present in the inner dental epithelium at the early bell stage, which was substituted by CK 19 at the late bell stage when the ameloblasts were fully differentiated (13). In the present case, islands of odontogenic epithelium demonstrating positivity for CK 14 and 19, which may occur in order to aid in the diagnosis and demonstrate a terminal odontogenic differentiation of the epithelium of ossifying OF. This result was also demonstrated by Eversole *et al.* (4).

Considering the histological differential diagnosis, this lesion showed the typical fibroosseous microscopic features of an ossifying fibroma. This marked feature probably induces a misdiagnosis of a fibroosseous lesion after incisional biopsy. Therefore, immunohistochemical studies should be performed to clarify the diagnosis when the odontogenic epithelium has been associated with ossifications. The OF is considered a rare lesion, but information suggests that it can also be considered a distinct lesion. It is important to highlight that a review of the prior literature demonstrated only four cases of ossifying variants of OF, including this present case report. Therefore, it is important to note that new case reports regarding OF with ossifications are important in an attempt to better define this rare OF variant and describe its clinical, radiographic, and histological features, as well as its biological behavior.
